# Octacosanol restores stress-affected sleep in mice by alleviating stress

**DOI:** 10.1038/s41598-017-08874-2

**Published:** 2017-08-21

**Authors:** Mahesh K. Kaushik, Kosuke Aritake, Atsuko Takeuchi, Masashi Yanagisawa, Yoshihiro Urade

**Affiliations:** 1 Department of Molecular Sleep, International Institute for Integrative Sleep Medicine (WPI-IIIS), University of Tsukuba, 1-1-1 Tennodai, Tsukuba, Ibaraki 305-8575 Japan; 2 Department of Molecular Genetics, International Institute for Integrative Sleep Medicine (WPI-IIIS), University of Tsukuba, 1-1-1 Tennodai, Tsukuba, Ibaraki 305-8575 Japan; 3 Kobe Pharmaceutical University, Higashinada, Kobe 658-8558 Japan

## Abstract

Octacosanol, a component of various food materials, possesses prominent biological activities and functions. It fights against cellular stress by increasing glutathione level and thus scavenging oxygen reactive species. However, its anti-stress activity and role in sleep induction remained elusive. We hypothesize that octacosanol can restore stress-affected sleep by mitigating stress. Cage change strategy was used to induce mild stress and sleep disturbance in mice, and effects of octacosanol administration on amount of sleep and stress were investigated. Results showed that octacosanol did not change rapid eye movement (REM) or non-REM (NREM) sleep compared to vehicle in normal mice. However, in cage change experiment, octacosanol induces significant increase in NREM sleep at doses of 100 and 200 mg/kg (75.7 ± 14.9 and 82.7 ± 9.3 min/5 h) compared to vehicle (21.2 ± 5.1 min/5 h), and decreased sleep latency. Octacosanol induced sleep by increasing number of sleep episodes and decreasing wake episode duration. Plasma corticosterone levels were significantly reduced after octacosanol (200 mg/kg) administration, suggesting a decrease in stress level. Octacosanol-induced changes in sleep-wake parameters in stressed-mice were comparable to the values in normal mice. Together, these data clearly showed that, though octacosanol does not alter normal sleep, it clearly alleviates stress and restore stress-affected sleep.

## Introduction

Octacosanol is a long-chain aliphatic alcohol extracted from wheat germ oil, rice bran oil, sugar cane along with its abundance in beeswax^1^. It is an antioxidant and has been reported to be effective in an animal model of parkinsonism^2–4^. Octacosanol is a major constituent of policosanol, a mixture of long-chain aliphatic alcohols. Studies using policosanol with 50–60% as octacosanol, affects lipid metabolism, reduces platelet aggregation^5^, showed antiulcer^6^ and anti-inflammatory activity^7, 8^. Policosanol also effectively decreases weight of the adipose tissue^9^ and inhibits cholesterol biosynthesis^10^. Octacosanol reduced liver injury by increasing glutathione (GSH) levels^3^, and increased GSH contributes to stress tolerance^11^.

Insomnia and other sleep disorders such as restless leg syndrome leads to brief sleep disturbances those in long-term results in chronic sleep deprivation. Sleep deprivation results in elevated levels of corticosterone, a marker of stress^12–14^, and stress is one of the major factors that results in sleep disruption^15, 16^. Synthetic drugs currently available for insomnia does not address stress component. Currently available insomnia drugs produce imbalance in neurotransmitters, leading to adverse effects and even dependency^17^.

In today’s world, where ever-changing environment and demanding job work enforces stress in humans, maintaining healthy lifestyle is a great challenge, hence, identification of bioactive compounds from food materials and plants has become a highly active area of pharmaceutical research, partly, because plant-based therapy is safer compared to synthetic drugs. Such compounds have been found effective in the treatment of various conditions, including anxiety, pain, and inflammation. Octacosanol is a promising compound due to its effects on central nervous system, and also because it increases GSH levels and thus could influence stress, we aimed to elucidate its effect on sleep and stress in mice. Therefore, we hypothesize that octacosanol administration alleviate stress and restores stress-affected sleep.

## Results

### Octacosanol did not induce sleep in normal mice

**Figure 1:**
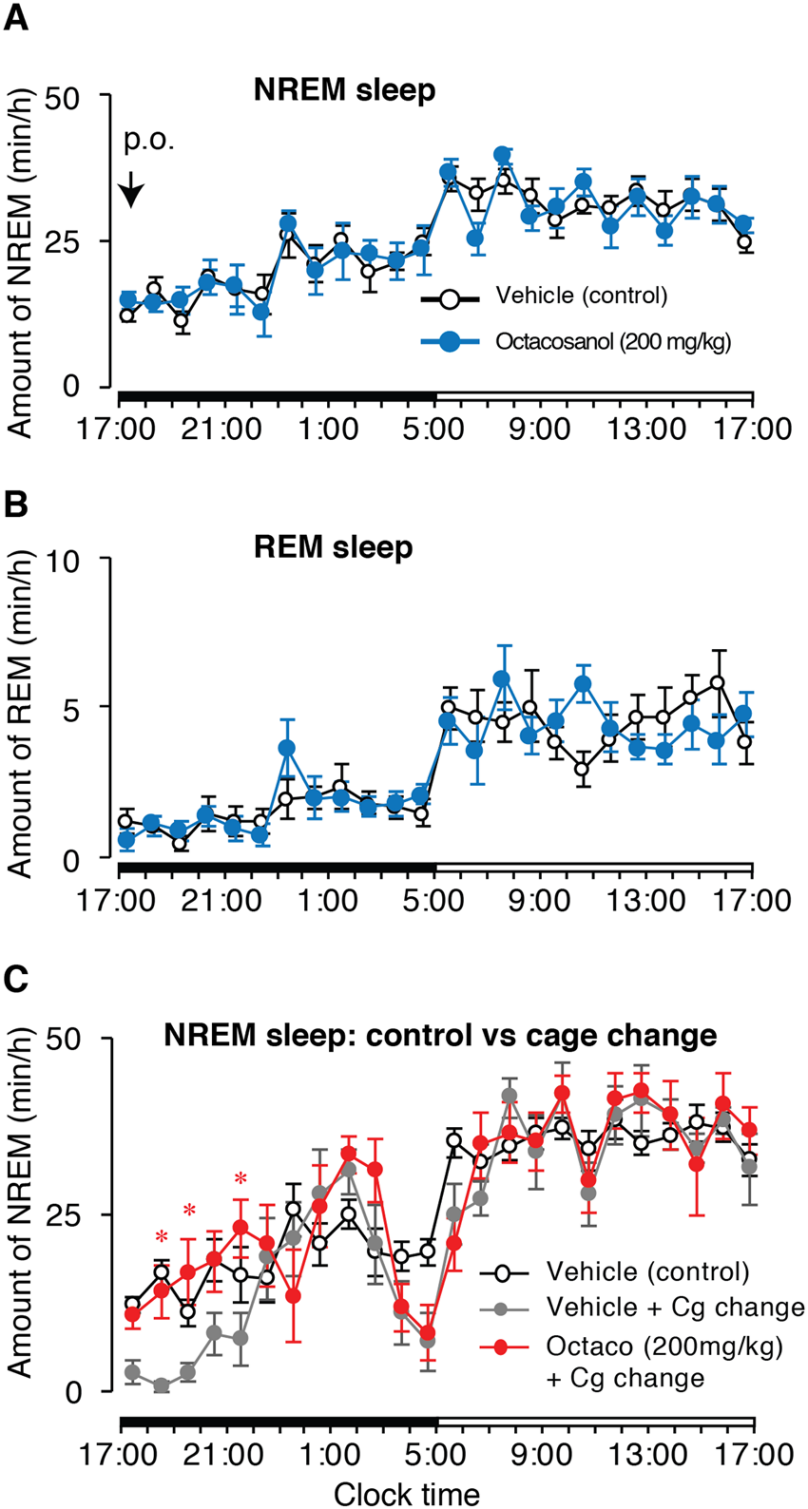
Effect of octacosanol oral administration in mice on sleep-wakefulness. Hourly plots of NREM (**A**) and REM (**B**) sleep after oral administration of vehicle (open circles) or octacosanol (blue circles). (**C**) Hourly plots of NREM sleep in mice after *p*.*o*. administration of vehicle (gray circles), octacosanol (red circles) with cage change, and control (vehicle with no cage change; open circles). Black and white horizontal bars indicate 12-h dark and 12-h light period. Data presented as mean ± SEM; n = 5–8; *p ≤ 0.05 vs veh + cg change by using two-way ANOVA. Cg: cage; octaco: octacosanol.

Octacosanol was administered in mice orally at the onset of dark period (17:00 h) and sleep was quantified over 24 h following *per os* (*p*.*o*.) administration. Time course changes showed that rapid eye movement (REM) or non-REM (NREM) sleep, and wake remain unchanged between the vehicle and octacosanol treatment (n = 8; Fig. 1A,B). This data clearly showed that octacosanol was not able to induce sleep in normal mice.

### Octacosanol induces significant amount of NREM sleep in mildly stressed mice

Next, we adopted the cage change strategy, whereby, mice were placed into a clean cage to induce constant wake for more than one hour^18^. Result showed that octacosanol induced significant amount of NREM sleep in mice after cage change (mild stress) compared to the vehicle. Further, we compared octacosanol-induced sleep (with cage change) to control (vehicle only; no cage change), and found that octacosanol treatment induced-NREM sleep in sleep-disturbed mice was equal to normal amount of NREM sleep in control animals (no cage change; n = 5–8; Fig. 1C). Here onwards, all the results explained were obtained by using cage change protocol.

### NREM sleep was increased and sleep latency was decreased, dose-dependently by octacosanol administration in mice

**Figure 2:**
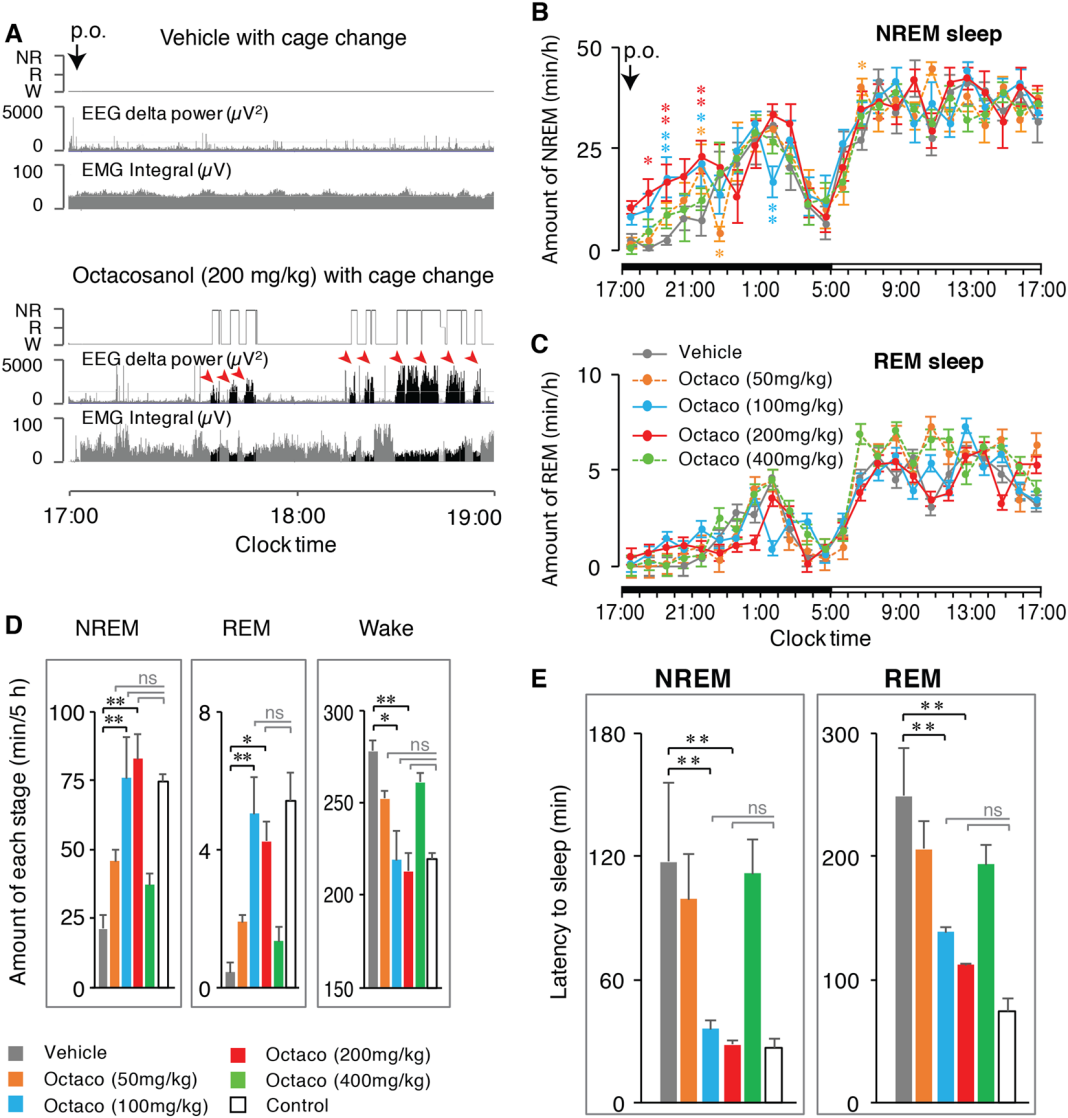
Oral administration of octacosanol dose-dependently increased NREM sleep and decreased wakefulness in mice. (**A**) Typical examples of EEG delta power (0.5–4 Hz), EMG integral, and hypnograms of a mouse after *p*.*o*. administration of vehicle or octacosanol. Hypnograms represent concatenated 10-sec epochs of EEG/EMG activity, scored as wake, REM, and NREM sleep. Two hours after *p*.*o*. administration are shown. Wake, REM are shown in gray while NREM sleep shown in black. Arrowheads shows increase in delta power. (**B**,**C**) Hourly plots of NREM and REM sleep in wild-type mice after oral administration of vehicle (gray circles) and various doses of octacosanol (color circles). Black and white horizontal bars indicate 12-h dark and 12-h light period. Data presented as mean ± SEM; n = 5; *p ≤ 0.05, **p ≤ 0.01, vs vehicle, by using two-way ANOVA followed by Least Square Difference (LSD) post hoc test. (**D**) Total amount of wake, REM, and NREM sleep over 5 h during dark period, (**E**) changes in NREM and REM sleep onset latency after vehicle (gray bar) and various doses of octacosanol (color bars) administration. Open bars (**D**,**E**) represent control (whereby vehicle was administered while animals remained in their home cages). Data presented as mean ± SEM; n = 5–8; *p ≤ 0.05, **p ≤ 0.01, vs vehicle, by using one-way ANOVA followed by Scheffe post hoc test. Octaco: octacosanol, ns: not significant.

Figure 2A shows typical electroencephalogram (EEG) delta power traces, electromyogram (EMG) integral and hypnograms of a mouse after *p*.*o*. administration of vehicle and octacosanol (200 mg/kg). Whereas the animal remained mainly awake after vehicle administration, octacosanol-treated animals fell asleep within 30 minutes. EEG trace during octacosanol treatment showed clear increase in delta power (arrowheads), characteristic to NREM sleep. However, vehicle-treated animal did not show such bouts of increase in delta power. EMG integral power decreased corresponding to the increase in EEG delta power (Fig. 2A). Time course analysis of hourly amounts of NREM and REM sleep revealed that octacosanol at the doses of 100 and 200 mg/kg, but not 50 and 400 mg/kg, increased NREM sleep significantly, at least for up to 5 h (n = 5; Fig. 2B). Hourly data of REM sleep did not show appreciable changes (Fig. 2C). Octacosanol administration increased NREM sleep dose-dependently from 21.2 ± 5.1 min/5 h after vehicle administration to 45.7 ± 4.2 (*p* = 0.413), 75.7 ± 14.9 (*p* = 0.002), 82.7 ± 9.3 (*p* = 0.000) and 37.1 ± 4.5 (*p* = 0.819) min/5 h, and decreased wake concomitantly from 278.4 ± 5.4 min/5 h after vehicle to 252.4 ± 4.0 (*p* = 0.331), 219.2 ± 15.8 (*p* = 0.012), 213.0 ± 9.7 (*p* = 0.000) and 261.6 ± 4.9 (*p* = 0.770) min/5 h after 50, 100, 200 and 400 mg/kg, respectively, after octacosanol administration during dark phase. However, NREM was significantly high only at 100 and 200 mg/kg (Fig. 2D). Total amount of REM sleep over 5 h also showed significant increase from 0.4 ± 0.3 min/5 h after vehicle administration to 1.9 ± 0.2 (*p* = 0.853), 5.0 ± 1.1 (*p* = 0.008), 4.2 ± 0.6 (*p* = 0.046) and 1.3 ± 0.4 (*p* = 0.977) min/5 h after 50, 100, 200 and 400 mg/kg octacosanol administration, respectively. Octacosanol dose-dependently decreased the latency to NREM sleep from 117 ± 38.06 min after vehicle to 99.2 ± 22.25 (*p* = 0.516), 36.10 ± 4.15 (*p* = 0.007) and 28.50 ± 1.81 (*p* = 0.004), respectively, after 50, 100 and 200 mg/kg octacosanol administration. Octacosanol at the dose of 400 mg/kg did not change sleep latency (111.6 ± 16.8 min; *p* = 0.002). Similar changes in REM latency were observed (Fig. 2E). All sleep-wake parameters were indistinguishable from control (Fig. 2D,E).

### Octacosanol induces sleep by increasing number of NREM episodes and by decreasing wake episode duration

**Figure 3:**
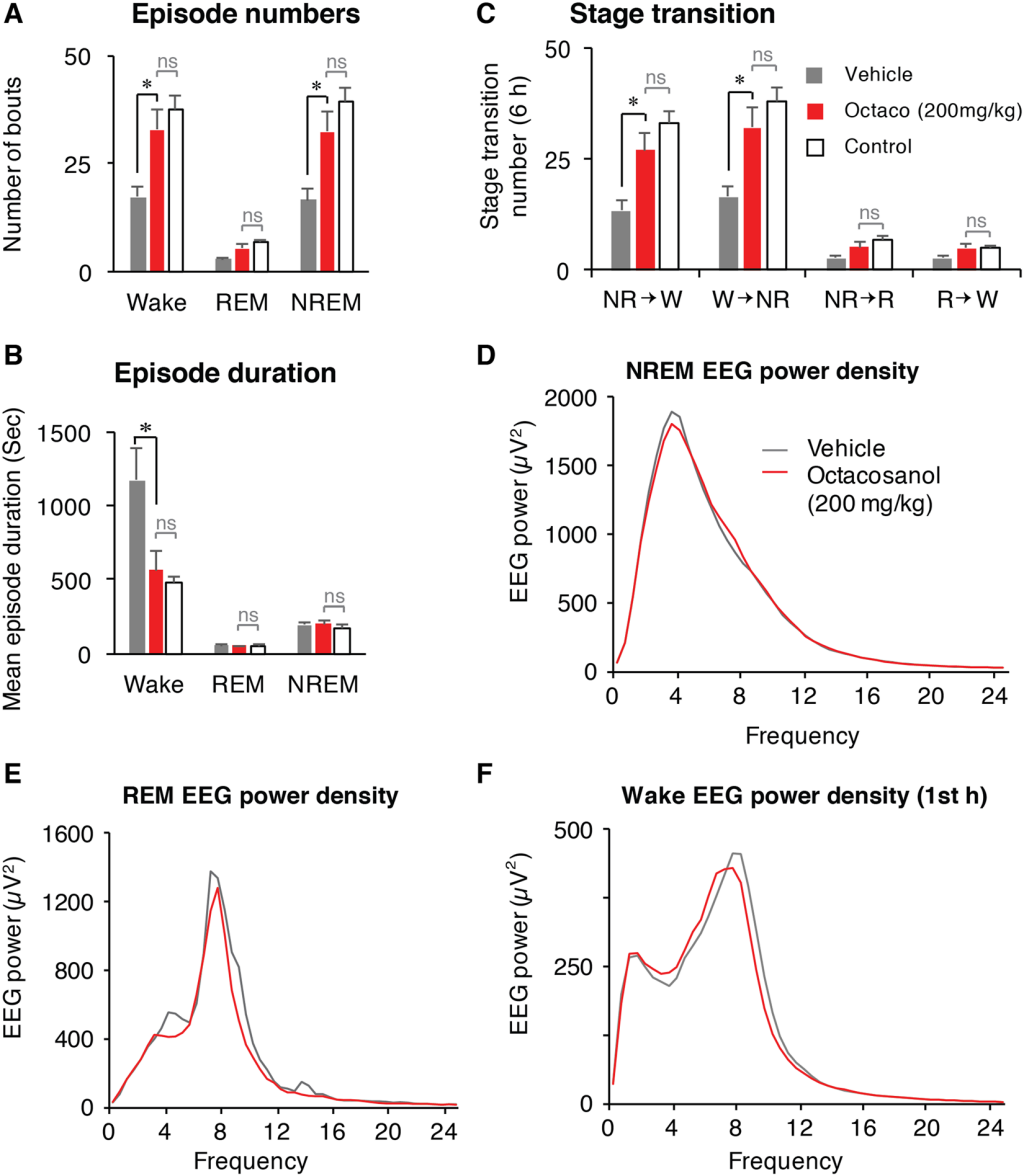
Changes in sleep architecture after octacosanol administration in mice. Graphs show qualitative analysis thus sleep architecture following *p*.*o*. administration of vehicle (gray bars), octacosanol (200 mg/kg; red bars), and control (open bars) during initial 6 h of dark phase. Graphs represent changes in number of episodes (bouts; **A**), mean duration (**B**) of wake, REM, and NREM sleep. (**C**) Graph shows stage transition from NREM to wake (NR→W), wake to NREM (W→NR), NREM to REM (NR→R) and REM to wake (R→W). (**D**–**F**) Graph shows the EEG power density of NREM (**D**) and REM (**E**) sleep over 6 h dark phase, and wake (**F**) over 1 h following vehicle (gray line) and octacosanol (red line) administration in mice. Data presented as mean ± SEM; n = 5–8; *p ≤ 0.05, vs vehicle, by using one-way ANOVA followed by Scheffe post hoc test.

The sleep-wake architecture and quality were evaluated by calculating episode numbers, episode duration, and stage transitions (n = 5). The episode number of NREM sleep (32.4 ± 4.46; *p* = 0.027) and wake (32.8 ± 4.59; *p* = 0.027) were significantly increased after treatment with octacosanol (200 mg/kg) compared to vehicle (16.6 ± 2.38 and 17.2 ± 2.29, respectively; Fig. 3A). Mean duration of wake episodes (564.0 ± 125.72 sec/episode; *p* = 0.020) was also decreased compared to vehicle (1172.4 ± 216.11 sec/episode; Fig. 3B). Octacosanol increased stage transition from wake-to-NREM (32.0 ± 4.51; *p* = 0.029) and NREM-to-wake (27.0±3.86; *p* = 0.033), compared to the vehicle (16.6 ± 2.38 & 13.4 ± 2.25, respectively; Fig. 3C). The value of these parameters were comparable to control values (Fig. 3A–C). To check the quality of octacosanol-induced sleep, power density was calculated. NREM and REM EEG power densities during 6 h dark phase were indistinguishable between vehicle and octacosanol treatments (Fig. 3D,E), indicating that octacosanol administration induced physiological sleep without affecting EEG power density. Similarly, EEG power density of wake during 1^st^ hour after administration was indifferent between two administration. Since, most data obtained after octacosanol administration and cage change is comparable to control values, our data strongly suggests that octacosanol restored sleep to control levels.

### Octacosanol effectively decreased blood plasma corticosterone, a marker of stress

**Figure 4:**
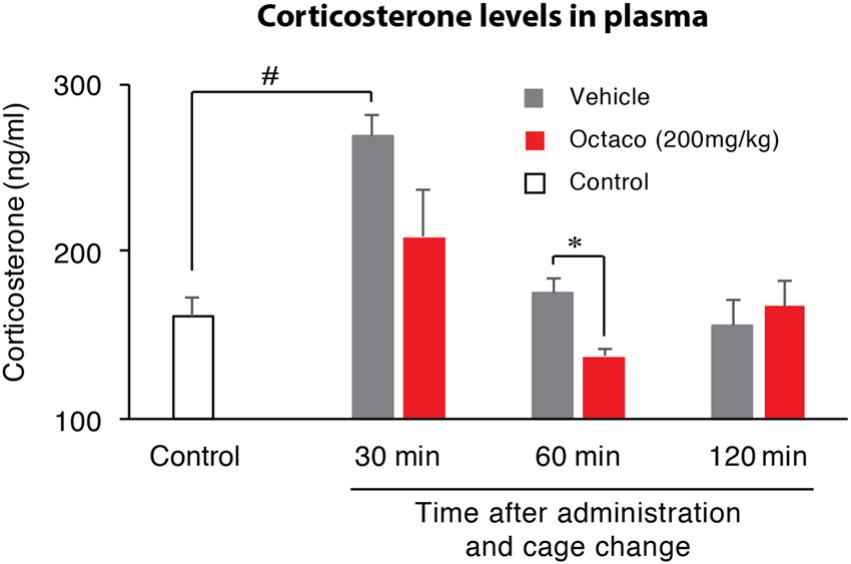
Changes in blood corticosterone levels (stress) after octacosanol administration in mice. Graph shows blood plasma corticosterone levels, after vehicle (gray bars) and octacosanol (200 mg/kg; red bars) administration in mice at various time points. Open bar represents control (no cage change). Data presented as mean ± SEM; n = 4–5; ^#^p ≤ 0.05 vs control, by using one-way ANOVA followed by Scheffe post hoc test, and *p ≤ 0.05 vs vehicle by using paired t-test. Octaco: octacosanol, ns: not significant.

The changes in stress level were studied following octacosanol or vehicle administration and cage change in mice. Blood samples were collected for estimation of corticosterone levels at 30, 60 and 120 min after administration and cage change. Results showed that cage change with vehicle administration increased plasma corticosterone levels (from 162.2 ± 11.16 to 269.25 ± 12.51 ng/ml; *p* = 0.000) compared to control (no cage change). This increase was attenuated by octacosanol administration at 30 min (from 269.25 ± 12.51 to 209.0 ± 27.53 ng/ml) and significantly reduced at 60 min (from 175.8 ± 7.72 to 137.4 ± 4.73 ng/ml; *p* = 0.029). Corticosterone levels were returned to control levels in octacosanol as well as vehicle administered mice after 2 h (n = 4–5; Fig. 4). Together this data clearly suggests that octacosanol mitigates stress in stress-affected mice and restores sleep.

## Discussion

In present study, we clearly showed that octacosanol administration mitigates the stress in mice, that was reflected in terms of restored NREM sleep. Moreover, our data clearly showed that all sleep-wake parameters returned to control levels after octacosanol administration in stressed mice. We achieved this goal by adopting cage change strategy, whereby, transferring mouse to a clean cage from its home cage, induces mild stress that keeps the mouse awake for more than one hour^18^. For the first time, these results demonstrated that octacosanol, which is a food component present in rice bran, sugarcane, and wheat germ oil, has stress lowering and sleep-inducing property. This data supports our hypothesis that octacosanol induces sleep by alleviating stress. Hence it restores stress-affected sleep.

Octacosanol is considered a safe compound because it is obtained from plants that are part of everyday food. A number of studies claimed that octacosanol exhibits a variety of biological activities in humans and animal models^3, 19^. The decrease in blood corticosterone level in current study is well supported by a previous study by Ohata *et al*. whereby, octacosanol treatment increased hepatic GSH concentration in CCl_4_-intoxicated rats^3^, however, its effect on brain GSH is unknown. GSH is primarily known for its scavenging function against reactive oxygen species, protection against biotic stress^20^, and detoxification of endogenous metabolic products including lipid peroxides and oxidative stress^21^. Taken together, this information and current decrease in corticosterone level, it can be logically argued that stressed-mice were unable to sleep, however, octacosanol restore normal sleep in these mice by reducing stress. It is well known that sleep and stress has inverse relationship because, disrupted sleep increases stress and health risks^22^, and increased stress affects the quality and quantity of sleep in mice^15, 18^ and in humans^16^.

The decrease in sleep latency in our study strongly suggests that octacosanol quickly mitigates the stress and results in reduced time to fall asleep in stressed-mice. Sleep regulation has two components *viz* sleep generation, signified by number of NREM episodes, and sleep maintenance, reflected by NREM episode duration. Octacosanol induced sleep by affecting sleep generation mechanism, suggested by increased NREM frequency, that also resulted in decreased wake episode duration. Octacosanol administration did not alter the NREM EEG power density, implying that quality of sleep was not affected, and that octacosanol-induced sleep was physiological. This is critically important because synthetic drug used for insomnia, such as benzodiazepines affect the quality of sleep and reduced EEG power^23^, suggesting, octacosanol can be a better alternative to currently available drugs because it promotes physiological sleep and combats stress.

Octacosanol supplements are widely used by humans and found to be effective for various conditions, some of which are supported by scientific studies. Despite it’s enormous use, almost nothing is known about its mechanism of action, its brain-blood-barrier penetrability or target brain region or neural types. Based on our findings, it can logically be argued that octacosanol directly or indirectly acts on the hypothalamic–pituitary–adrenal (HPA) axis to lower the stress level by decreasing the corticosterone secretion. This reduced stress level results in normalization of physiological functions such as sleep. However, Wang *et al*. showed that protective effects of octacosanol against Parkinson might be mediated by blocking the phosphorylation of p38MAPK and JNK on the signal transduction^24^, more work needs to be done to understand its mechanism of action.

Insomnia and poor quality of sleep results in chronic sleep loss that is associated with various other sleep and metabolic disorders. In today’s world, majority of population experience lifestyle related or other types of stress, a major factor affecting sleep quality and quantity. For the first time, we demonstrated that octacosanol is a potent anti-stress compound with sleep inducing potential. Being a natural compound, and part of food materials, are advantages over synthetic drug and hence it can be assumed that octacosanol may be devoid of side effects or adverse reactions to human body. Hence, we strongly suggest that octacosanol could be used as therapy for stress-induced insomnia.

## Materials and Methods

### Animals

Experiments were performed on male C57BL/6 mice weighing 24–30 g (11–13 weeks; n = 4–8 each group). C57BL/6 mice were obtained from SLC (Hamamatsu, Japan). Mice were housed in an insulated sound-proofed recovery chamber maintained at an ambient temperature of 23 ± 0.5 °C with a relative humidity of 50 ± 5% on an automatically controlled 12-h light/dark cycle (light on at 0500; illumination intensity above 100 lux). Mice were left undisturbed in this chamber to acclimatize to the new environment for 5–7 days before any procedure was performed. Mice had free access to food and water. Experimental protocols were approved by the University of Tsukuba Animal Ethics Committee, and all procedures and methods were performed in accordance with the relevant guidelines and regulation laid down by animal ethics committee (Animal ethical approval number; 16086). Every effort was made to minimize the number of animals used as well as any pain and discomfort.

### Surgery

Under pentobarbital anesthesia (50 mg/kg, intraperitoneally) mice were chronically implanted with EEG and EMG electrodes for polysomnography, as previously described^25, 26^. Antibiotic and analgesic drugs were administered up to 5 days post-operatively and physical condition of animals was observed. After 8–10 days of postoperative recovery, the mice were placed in experimental cages for a 4-day habituation/acclimatization period and connected with counterbalanced recording leads. All mice that were subjected to EEG/EMG recordings received vehicle and various doses of drug treatment (17:00 h; onset of dark phase) on different days.

### EEG/EMG recording and analysis

Cortical EEG and EMG signals were amplified and filtered (EEG, 0.5–30 Hz; EMG, 20–200 Hz), then digitized at a sampling rate of 128 Hz, and recorded by using SleepSign software (Kissei Comtec, Nagano, Japan) as previously described^27^. Polysomnographic recordings were scored with automated analysis, off-line, in 10-s epochs by SleepSign software, using standard criteria^28, 29^. Briefly, for each epoch, the software calculated the EEG power density in the delta (0.5–4.0 Hz) and theta band (6.0–10 Hz), and the integrated EMG. Three vigilance states were determined based on EEG delta & theta values and EMG integral as follows: wake (high EMG and low EEG amplitude and high theta activity concomitant with highest EMG integral values), NREM sleep (low EMG and high EEG amplitude, high delta activity), and REM sleep (low EMG and low EEG amplitude, high theta activity). Epochs containing two different vigilance states within a 10 s epoch, were given the score of the predominant state. The defined sleep–wake stages were visually examined and corrected, wherever necessary. Further, we calculated sleep latency as a measure of time from drug/vehicle administration to the appearance of at least two consecutive NREM sleep episodes. Spectral analysis of EEG by fast Fourier transformation (FFT) was performed, and the EEG power densities of each 0.5- Hz bin were averaged by calculating the absolute power of each bin.

### Chemicals and pharmacological treatment

Octacosanol (Sigma-Aldrich) was administered *per os* (*p*.*o*.) in mice at concentration i.e. 50, 100, 200 and 400 mg/kg, on separate days. Octacosanol was suspended in 20% Vit-E TPGS (D-α-Tocopherol polyethylene glycol 1000 succinate). All the administrations were done at the onset of dark phase (17:00 h). At least 2-day washout period was given between two administrations. Vit-E TPGS was prepared by dissolving 20 g in 75 ml of double-distilled H_2_O on magnetic stirrer overnight with mild heat, final volume was adjusted to 100 ml. The drugs were suspended in vehicle immediately before use and administrated orally (10 ml/kg) by using a disposable 1 ml syringe and gavage needle (0.9 mm diameter). At the same time, animals housing cages were exchanged with new cages having fresh bedding and food in order to increase the wakefulness. Scientific studies where octacosanol alone was used in humans are limited. Keller *et al*., studied the use of 30 mg/day (10 mg each with 3 principle meals) for 4 weeks in human females and did not report any adverse effect^30^. Most studies on humans used policosanol, with octacosanol as major constituent and suggested a safer dose of 10–20 mg/day for humans^31, 32^. The dose used in this study is much higher compared to suggested human dose, however, a much higher dose (500 mg/day) of policosanol continuously for 1-year was found to be safe in rats^33^.

### Blood sampling and corticosterone measurement

It has been reported that placing a mouse to a new clean cage result in mild stress which is reflected as an increase in blood corticosterone level^18^. To test the degree to which octacosanol decrease stress in mice after they were placed in new clean cage environment, we measured the circulating corticosterone in blood. The mice were administered with vehicle or octacosanol (200 mg/kg) and placed in clean cages. Blood sampling was performed by cardiac puncture under deep anesthesia at 30, 60 and 120 min after the mice were placed in clean cages. Each blood sampling was conducted within 2 min, which is rapid enough to ensure that the stress imposed in the blood sampling procedure did not affect corticosterone levels in plasma. Each mouse was used only once and blood was collected in the EDTA (0.05%)-coated syringe and collected in 1.5 ml tubes. The samples were immediately centrifuged at 10,000 rpm for 15 min at 4 °C, plasma samples were collected in sterile tubes and frozen at −80 °C until used for estimation. Plasma corticosterone was measured using HPLC.

### Statistical analysis

All data were expressed as the mean ± SEM. The statistical significance of time-course data for sleep-wake profiles was calculated by using two-way ANOVA (repeated measures). NREM REM and wake EEG power density were analyzed by using paired t-tests. For the dose-response effects on sleep latency, amounts of NREM sleep, REM sleep, and wake, number of sleep-wake bouts, bout duration, stage transition, one-way ANOVA followed by the Least Square Difference (LSD) posthoc test was used. In all of the cases, p < 0.05 was considered as significant.

### Data Availability

All data generated or analysed during this study are included in this published article.
